# Deciphering Bacterial Chemorepulsion: The Complex Response of Microbes to Environmental Stimuli

**DOI:** 10.3390/microorganisms12081706

**Published:** 2024-08-18

**Authors:** Ruixin Fu, Haichao Feng

**Affiliations:** 1 School of Biology and Food, Shangqiu Normal University, Shangqiu 476000, China; ruixinfu2022@163.com; 2 College of Agriculture, Henan University, Kaifeng 475004, China; 3 Food Laboratory of Zhongyuan, Henan University, Luohe 462300, China

**Keywords:** chemorepulsion, chemorepellent, plant growth-promoting rhizobacteria (PGPR), methyl-accepting chemotaxis protein (MCP), biphasic chemotaxis

## Abstract

Bacterial motility relying on flagella is characterized by several modes, including swimming, swarming, twitching, and gliding. This motility allows bacteria to adapt remarkably well to hostile environments. More than 50% of bacteria naturally contain flagella, which are crucial for bacterial chemotaxis motility. Chemotaxis can be either positive, where bacteria move towards a chemical source, or negative, known as chemorepulsion, where bacteria move away from the source. Although much is known about the mechanisms driving chemotaxis towards attractants, the molecular mechanisms underlying chemorepulsion remain elusive. Chemotaxis plays an important role in the colonization of the rhizosphere by rhizobacteria. Recently, researchers have systematically studied the identification and recognition mechanisms of chemoattractants. However, the mechanisms underlying chemorepellents remain unclear. Systematically sorting and analyzing research on chemorepellents could significantly enhance our understanding of how these compounds help probiotics evade harmful environments or drive away pathogens.

## 1. Introduction

Motility is important not only for bacterial survival but also for the establishment of symbiotic relationships. Bacterial motility primarily involves mechanisms driven by flagella (swarming and swimming), type IV pili (twitching), and other modes (gliding, sliding, etc.). These movements occur across various environments, including solid surfaces (swarming, twitching, gliding, and sliding) and liquid mediums (swimming) [1]. Among these, flagellar motility is particularly important for bacterial adaption and is present in more than 50% of the known bacterial species [2].

Bacterial chemotaxis refers to the movement response of bacteria to a concentration gradient of signaling substances, which mainly control movement behavior by regulating the direction of flagellar rotation. This process enables microorganisms to target favorable stimuli (chemoattractants) or avoid harmful stimuli (chemorepellents). The study of chemotaxis dates back to the 1880s, with Thomas Engelmann and Wilhelm Pfeffer discovering that the movement of bacteria is directional rather than random [3,4]. In 1960, Julius Adler was the first to systematically investigate the molecular mechanism underlying chemotaxis in *Escherichia coli* and proposed that bacterial chemotaxis is controlled by chemoreceptors located on the surface of cells [5]. Shortly thereafter, Ordal conducted extensive studies one chemotaxis in *Bacillus subtilis*, comparing the differences between *B. subtilis* and *E. coli* chemotaxis, providing a reference for understanding the molecular mechanisms across different bacterial species [6,7].

Chemotaxis plays a crucial role in various processes, including colonization [8], biofilm formation [9], nitrogen fixation [10], pollutant degradation [11], pathogenesis [12], and bacterial migration in soil [13]. Most bacteria regulate the direction of mobility by sensing and responding to concentration gradients of substances in their environment [14]. For example, a dynamic diffusion gradient of chemoattractants in the environment prompts bacteria to continue straight swimming, while chemorepellents increase the frequency of tumbling to help bacteria escape from unfavorable conditions [15]. Through a combination of tumbling and smooth swimming, bacteria gradually navigate across a concentration gradient of substances. Although research on chemoattractants has progressed rapidly, the identification and characterization of chemorepellents remain relatively limited. However, with the deepening of studies on bacteria–plant interactions, the role of chemorepellents is becoming increasingly prominent, particularly with regard to their ability to help probiotics evade unpleasant environments or repel pathogens.

**Figure 1:**
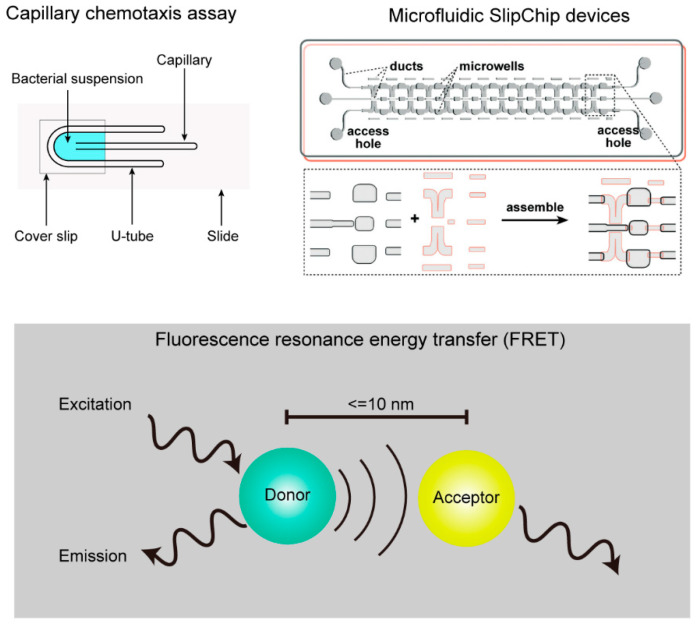
Bacterial chemotaxis assays, including the classic capillary chemotaxis assay for the quantitative experiment, the simple and reusable microfluidic SlipChip device for the high-throughput screening analysis, and the real-time detection of chemotactic signal changes by fluorescence resonance energy transfer.

Classic methods of studying chemotaxis include the capillary method [16], titration method [17], floating plate method [18], and the agarose capillary method [19]. With advancements in science and technology, and the development of new materials, research on chemotaxis has already entered a phase of rapid progress (Figure 1). To date, many high-efficiency detection methods are widely used to study and analyze bacterial chemotaxis, including simple and reusable microfluidic SlipChip devices [20], real-time detection of chemotactic signal changes using fluorescence resonance energy transfer (FRET) [21], and high-throughput rapid screening of chemotactic ligands by differential scanning fluorimetry (DSF) [22].

## 2. Molecular Mechanism of Bacterial Chemotaxis

### 2.1. Motile Patterns of Chemotactic Bacteria

Chemotaxis involves the movement of individual bacteria, guided by the rotation of their flagella. Most bacterial flagella, represented by *E. coli*, can rotate in two distinct ways, resulting in different types of movement; (i) counterclockwise rotation results in smooth swimming where the flagella bundle together, allowing the bacteria to move steadily in one direction, and (ii) clockwise rotation causes the flagella to disperse, leading to tumbling where the bacteria change direction randomly [23]. The chemotactic mechanisms of *E. coli* and *B. subtilis* are similar [7]. Bacteria control the rotation of flagella by sensing different gradient concentrations of chemoeffectors in the surrounding environment, which allows them to adjust the direction of their movement. Typically, in the absence of chemoattractants, bacteria swim smoothly in a straight line for a brief period before tumbling to change direction. When chemoattractants are detected, bacteria maintain their original direction and continue swimming. Conversely, when chemorepellents are sensed, they tumble to alter their direction and move away from the harmful stimuli.

### 2.2. Molecular Mechanism of Chemotaxis

Chemoreceptors, which can undergo methylation, are referred to as methyl-accepting chemotaxis proteins (MCPs). MCPs also function as signal sensors and are therefore sometimes called transducer-like proteins (TLPs). The chemotactic transduction pathway includes the following three parts: (i) signal recognition by MCPs, (ii) signal transduction from MCPs to flagellar motors, and (iii) adaptation to signals.

#### 2.2.1. Recognition of Chemotactic Signals

MCPs form a ternary complex with the histidine kinase CheA and the coupling protein CheW (or CheV). When chemotactic signals are present in the external environment, MCPs bind these signals and transmit them by changing the structure of proteins.

In general, MCPs contain five domains: (i) the recognition domain (extracellular), (ii) the transmembrane domain (TM helices), (iii) the connection domain (HAMP domain), (iv) the methylation region, and (v) the downstream signal domain [24]. The signal recognition domain, also known as the ligand binding domain (LBD), is responsible for binding chemoeffectors. Different bacterial species may have receptors with varying LBD types and topologies, and the number of chemoreceptors differs among species. Therefore, in-depth studies of the LBD are crucial for understanding how MCPs bind a wide array of chemoeffectors [14]. The methylation domain, which consists of two helical bundles with conserved glutamine and glutamate residues, undergoes methylation by the methyltransferase CheR and is demethylated by the methylesterase CheB, allowing bacteria to adapt to changes in the concentration of chemoeffectors. This domain is also a key feature for classifying proteins as MCPs [25].

To date, more than 80 types of LBDs have been identified, with many more yet to be discovered [26]. An analysis of 26,530 MCP sequences extracted from the Pfam 31.0 database, using the sequences of the MCP-specific methylation domain as templates, revealed that 15,872 sequences had known LBDs, while the remaining 10,658 sequences corresponded to unknown LBDs [14]. Among these MCPs, the three most common LBDs are the Cache (calcium channels and chemotaxis receptors), 4HB (four-helix bundle), and PAS (Per-Arnt-Sim). The Cache family, divided into single Cache (sCache) and double Cache (dCache), accounts for 33% of the total amount, with dCache being the most abundant family of LBDs in bacteria. The 4HB accounts for 31% and is commonly found in both bacteria and archaea [27]. The Tar and Tsr of MCPs in *E. coli* are the most thoroughly studied examples of these receptors [28]. The PAS domain, unlike the Cache and 4HB, is mainly present in prokaryotes [29]. Beyond these three major LBD types, there are over 30 additional kinds, although they collectively comprise less than 20% of the total LBDs. Therefore, the abundance of different LBDs varies greatly across different species.

The mechanisms by which the most abundant LBD in bacteria, dCache, senses small molecular signals have been extensively studied. For example, in the rhizosphere growth-promoting plant bacterium *B. velezensis* SQR9,the chemoreceptor McpA can sense a variety of chemoeffectors with different structures, including five amino acids (aspartate, glutamate, isoleucine, lysine and tyrosine), ten organic acids (malic acid, citric acid, oxalic acid, succinic acid, fumaric acid, phthalic acid, adipic acid, dehydroascorbic acid, glyceric acid and 3-hydroxypropionic acid) and five other substances (tert-butyl hydroxycarbamate, mannose, ribose, trehalose, and ribosyl alcohol) [20,30]. In *Campylobacter jejuni* 11168, the LBDs of chemoreceptors CcmL (Tlp3) and CcrG (Tlp11), both belonging to the dCache, also bind multiple ligands. CcmL can directly bind amino acids (isoleucine, lysine, and arginine), purines, and succinate [31]. CcrG, which has the first known sugar-binding dCache domain, directly binds galactose with a high affinity [32]. In *Pseudomonas aeruginosa* PAO1, a common conditioned pathogen in soil, the chemoreceptors PctA, PctB, and PctC all directly bind amino acids [33,34]. Similarly, *P. putida* KT2440 contains MCPs (McpA, McpH, McpU, and McpG) that directly bind 12 amino acids [9], purines [35], polyamines [9], and γ-aminobutyric acid (GABA) [36], respectively. In *Sinorhizobium meliloti* MV II-1, a nitrogen-fixing bacterium capable of mutual symbiosis with legumes, chemotaxis plays an essential role in establishing the symbiotic relationship. The chemoreceptor McpU in *S. meliloti* MV II-1 binds proline and mediates the bacterium’s chemotactic response toward its host plants [37]. In addition, *S. meliloti* McpX binds quaternary amines produced during seed germination, which can serve as plant nutrients, osmoprotection agents, and intercellular signaling molecules [38].

#### 2.2.2. Signal Transduction from MCPs to Flagellar Motors

**Figure 2:**
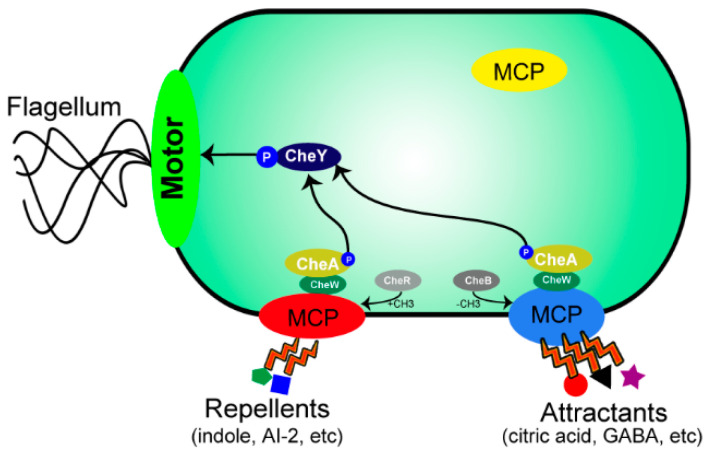
The mobility patterns of chemotaxis. General model of bacteria recruitment to the distinct signal sources through chemotaxis.

CheA and the response regulatory protein (CheY) constitute a two-component regulatory system that jointly regulates downstream chemotactic signal transduction [39]. These two proteins are central to the chemotaxis pathway and are conserved across bacterial species, with some variability [40]. For instance, in *E. coli*, the signal transduction from MCPs to flagellar motors occurs as follows (Figure 2). With the increase in chemoattractants or the decrease in chemorepellents, the conformation of the chemoreceptor changes, inhibiting the self-phosphorylation of CheA. This inhibition results in a decrease in the concentration of phosphorylated CheA (CheA-p) and subsequently, a decrease in the concentration of phosphorylated CheY (CheY-p). CheY-p interacts with the flagellar motor, changing either the rotational direction or speed of flagella rotation and inducing tumbling to move away from high concentrations of repellents or low concentrations of attractants [41,42]. In contrast, in *B. subtilis*, when MCPs bind to chemoattractants, the self-phosphorylation of CheA is activated, leading to an increase in the concentration of CheY-p, causing the flagella to rotate in the counterclockwise direction and direct swimming [7]. Additionally, in *E. coli*, the phosphoryl group from CheY-p is removed by the phosphatase CheZ [43], whereas in *B. subtilis*, this function is performed by the motor-associated protein FliY [24]. The coupling protein CheW is essential for this process. Without CheW, the entire signal transduction pathway is interrupted, resulting in the loss of chemotaxis [44].

#### 2.2.3. Adaptation to Signals

The ability of bacteria to respond to chemoeffectors depends on the regulation of adaptive mechanisms. The chemoreceptors bind to chemoeffectors and affect the kinase activity of CheA. Over time, the sensitivity and activity of chemotactic proteins change through a series of signal regulation steps, eventually reaching an equilibrium state [45]. In *E. coli*, adaptation is relatively simple, involving only one regulatory pathway, the methylation system. In this system, methyltransferase (CheR) and methylesterase (CheB) coregulate adaptation to the environment by covalently modifying MCPs. In contrast, *B. subtilis* employs three distinct adaptation mechanisms: the methylation system, the CheC-CheD-CheY-p system, and the CheV system.

Among the above three adaptation mechanisms in *B. subtilis*, the first is the methylation system. In this system, the chemotactic receptor is rapidly demethylated and then methylated in the presence of a chemotactic substance, for which the same process occurs when the chemoattractant is removed [46]. Moreover, methanol is produced by the addition or removal of chemoattractants (methanol is a product of demethylation catalysed by CheB) [47]. In addition, the level of methylation remains approximately constant, independent of the external concentration of the chemoattractants. In contrast, in *E. coli*, the adaptation is more straightforward as the chemoreceptor inhibits the kinase activity of CheA in the presence of chemoattractants. Over time, the chemoreceptor is methylated by CheR to build a CheA-active form; CheB then becomes phosphorylated by CheA, leading to the demethylation of chemoreceptors and the production of methanol [48].

The second adaptive system in *B. subtilis* involves coregulation by phosphatase (CheC) and deamidase (CheD). On the one hand, CheC hydrolyses CheY-p, reducing its concentration [24]. On the other hand, CheD converts the glutamine in the chemoreceptor to glutamic acid, making the receptor prone to methylation, which in turn promotes the self-phosphorylation of CheA and increases the concentration of CheY-p [49].

The third adaptive system is regulated by the coupling protein CheV. CheV comprises two domains, an N-terminal, a CheW-like linkage domain, and a C-terminal, a reaction regulatory domain, which are phosphorylated by CheA [50]. CheV connects MCPs and CheA, with the specific regulatory process functioning as follows: the autophosphorylation of CheA can regulate the phosphorylation of CheV, while CheV-p can inhibit the phosphorylation of CheA [7]. Some bacteria, such as *Helicobacter pylori*, which lack CheR and CheB, may rely on CheV as their sole adaptation mechanism [51].

## 3. Research Progress in Chemorepellents

**Figure 3:**
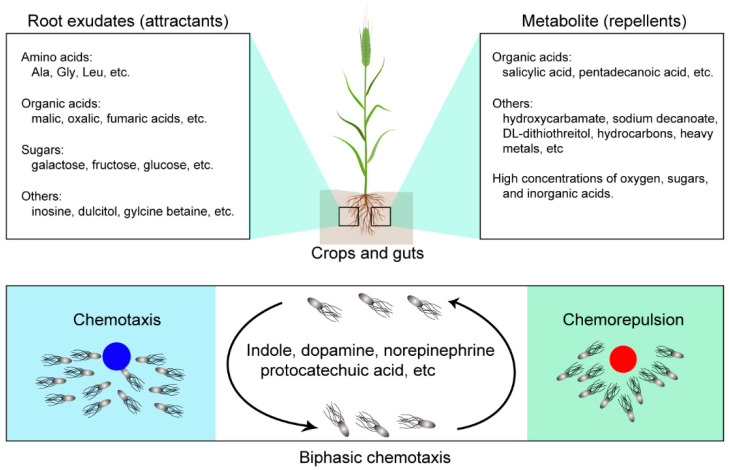
Common attractants and repellents in the crop’s rhizosphere or guts, and bacterial biphasic chemotactic response.

Research on bacterial chemotaxis has focused mainly on screening chemoattractants and identifying their corresponding chemoreceptors, while studies on chemorepellents and negative chemotaxis have been less extensive (Figure 3). Initial reports on chemorepulsion date back to the late 19th century and early 20th century, and researchers have found that bacteria can avoid certain substances, including salts, acids, bases, alcohols, and high concentrations of oxygen [52]. For example, Lederberg et al. reported that *Salmonella typhimurium* showed a chemorepulsion to phenol [53]. Clayton et al. found that *Rhodospirillum rubrum* showed a negative response to amino acids and salts [54]. *P. fluorescens* tends to avoid acids, bases, salts, and alcohols [55]. Additionally, *P. marinoides* has been reported to display an aversion to hydrocarbons and heavy metals [56]. *S. typhimurium* has also been observed to show a negative chemotaxis to insect repellents [57]. *P. aeruginosa* PAO1 has been shown to exhibit negative chemotaxis to trichloroethylene (TCE), tetrachloroethylene (PCE), chloroform, and dichloromethane [58]. Furthermore, *E. coli* has been shown to avoid high concentrations (more than 1 M) of sugars and inorganic acids [59].

There have been few reports on chemoreceptors that can bind both chemoattractants and chemorepellents simultaneously. Notable examples include CcmL in *C. jejuni* 11168 [31], PctA in *P. aeruginosa* PAO1 [60], and McpA in *B. velezensis* SQR9 [30]. CcmL directly binds amino acids (isoleucine, lysine, and arginine), purine, and succinate. In *C. jejuni* 11168, CcmL exhibits chemotaxis towards malic acid, fumaric acid, isoleucine, and purine, but tends to avoid succinic acid, thiamine, glucosamine, arginine, and lysine [31,32]. McpA in *B. velezensis* SQR9 shows a chemotactic response to 19 substances, such as aspartic acid, glutamic acid, malic acid, citric acid, and ribose, while showing chemorepulsion to hydroxycarbamate [20,30].

### 3.1. Chemorepellents in Pathogens

*E. coli* is a model organism for studying bacterial chemotaxis. It contains five MCPs—Tar, Tsr, Tap, Trg, and Aer—that mediate chemotaxis to amino acids, peptides, and sugars [61]. *E. coli* has been observed to avoid melatonin (a hormone) and spermidine (a polyamine) (Table 1). Further studies have revealed that the periplasmatic binding protein PotD binds spermidine, which is then recognized by the chemoreceptor Trg, albeit in low abundance [21]. The avoidance response likely helps bacteria evade high concentrations of hormones and polyamines in the intestine, thereby avoiding the antibacterial effects in the mucosal layer and enabling bacteria to locate the most suitable ecological niche for growth. In addition, *E. coli* shows obvious chemorepulsion to high concentrations of protocatechuic acid, which inhibits its growth. Conversely, *E. coli* shows strong chemotaxis towards low concentrations of protocatechuic acid [62], suggesting that chemorepulsion plays a dominant role in the chemotactic behaviour of *E. coli*. A combination of computer simulation and experiments led to the screening of various chemotactic ligands in *E. coli*. For example, *E. coli* exhibits an evasive response to 1-aminocyclohexane carboxylic acid, which directly binds to the chemoreceptor Tsr without the involvement of periplasmic binding proteins [63]. Site-directed mutagenesis experiments further demonstrated that Asn68 plays an important role in regulating chemotaxis. Notably, the chemotaxis response of the mutant strain to leucine shifted from negative to positive when Asn68 mutated to alanine [63].

Bacteria can exhibit biphasic chemotaxis to certain compounds. For instance, *E. coli* shows strong and rapid chemorepulsion to indole at concentrations below 1 mM via the chemoreceptor Tsr (Table 1). However, at concentrations above 1 mM, *E. coli* shows a time-dependent chemotaxis mediated by the chemoreceptor Tar, characterized by a long and slow reaction from the rejection reaction. This suggests that indoles, as gut metabolites, can either repel and attract foreign invasive bacteria depending on their adaptation to indoles [64]. *E. coli* also shows a biphasic chemotactic response to other compounds such as dopamine, protocatechuic acid, and norepinephrine. At dopamine concentrations below 1 mM, *E. coli* tends to exhibit chemorepulsion, while at concentrations of 10 mM, positive chemotaxis is observed. Similarly, *E. coli* exhibits chemotaxis towards protocatechuic acid at concentrations below 50 μM and towards norepinephrine below 1 mM, but it shows chemorepulsion at higher concentrations [21]. Additionally, *E. coli* demonstrates a negative response to leucine and valine at high concentrations and positive chemotaxis at low concentrations, possibly as an evolved mechanism to avoid a toxic level of these non-catabolized amino acids [65]. Furthermore, *B. subtilis* also displays biphasic chemotaxis; it is attached to low micromolar concentrations of phenol through the chemoreceptors HemAT and McpC, but phenol acts as a chemorepellent at higher millimolar concentrations via McpA [66].

To adapt to the host’s intestinal environment, bacteria have evolved specific mechanisms to sense and respond to stimuli in hostile conditions. *H. pylori*, a Gram negative bacterium, colonizes the stomach mucosa in humans and is associated with gastritis, peptic ulcers, and other diseases. *H. pylori* exhibits chemorepulsion to the six bile acids (taurocholic acid, taurodeoxycholic acid, glycholic acid, glycine deoxycholic acid, glycine deoxycholic acid, and taurocholic acid) and two amino acids (aspartic and glutamic acid) (Table 1). Among these, *H. pylori* shows the strongest avoidance to taurine and taurodeoxycholic acid [67]. Bile acid acts as bacteriocide against *H. pylori* [68]. Autoinducer-2 (AI-2) is not only an important quorum sensing factor, but also plays a significant role in regulating the spatial organization and diffusion of biofilms [69]. Therefore, identifying the chemotactic receptors that respond to AI-2 in *H. pylori* is essential. It has been reported that *H. pylori* uses AI-2 as a repellent, mainly through two newly identified periplasm-binding proteins, AibA and AibB [70], which then interact with the chemoreceptor TlpB [71]. *H. pylori* also shows a certain avoidance response to low pH [72] and reactive oxygen species [73]. Studying *H. pylori* chemotaxis is highly important as this bacterium can colonize the gastric mucosa and regulate its cell density. Similarly, *Ligilactobacillus agilis* BKN88 displays chemorepulsion to intestinal-borne compounds, such as low pH, organic acids, and bile salts. Unlike non-motile microorganisms, motile bacteria use chemotaxis to escape adverse environments rather than improving stress tolerance and resistance [74].

D-type amino acids (DAAs) are widely distributed across various organisms, particularly in bacteria. In general, DAAs do not directly participate in protein synthesis, but atypical DAAs perform unique functions in bacterial physiology. For example, DAAs are crucial components of bacterial cell walls (peptidoglycan), they regulate the bacterial surface charge and autolysin activity, influence biofilm depolymerization, affect bacterial growth, and determine the ecological niche of bacteria [75]. *Vibrio cholerae* contains at least 45 functionally redundant chemoreceptor genes, including Mlp24 and Mlp37, which are involved in the sensing of amino acids [76,77]. *V. cholerae* mediated an avoidance response to DAAs (D-Arg and D-Asp), mainly through the chemoreceptor MCP VC1313. To further characterize the precise mechanism underlying ligand specificity, seven point mutants of the binding sites (N43A, D47A, T48A, T105A, W107A, E111A, and N176A) were constructed, five of which were necessary for the rejection response to D-Arg. Therefore, chemorepulsion to DAA may serve as an important indicator for assessing the habitability of a plant’s ecological niche.

As the navigational system guiding bacterial movement, chemotaxis plays an important role in pathogenic virulence. Therefore, targeting the rejection of bacterial pathogens through chemotaxis inhibition may offer a promising therapeutic strategy for disease prevention. Systematic screening of chemorepellents that directly bind chemoreceptors could provide valuable insights for the rational design of bacteriocides to control the chemotaxis of pathogens.

### 3.2. Chemorepellents in PGPR

Bacteria can avoid not only the toxic and harmful compounds, but also the organic acids and alcohols found in root exudates. Feng et al. used a microfluidic SlipChip device to systematically screen the chemoeffectors of *B. velezensis* SQR9 against 99 compounds in cucumber root exudates. Ultimately, SQR9 exhibited negative chemotaxis to only five compounds (Table 1). Further studies showed that McpA played a major role in the chemotactic response to hydroxycarbamate, while McpB was crucial in preventing the reaction to salicylic acid and sodium decanoate. TlpA and TlpB were found to be dominant in mediating chemorepulsion to DL-dithiothreitol and pentadecanoic acid, respectively [20]. This study holds significant potential for the targeted regulation of bacterial motility and colonization.

SQR9 strongly promoted chemotaxis towards glutamic acid, citric acid, and mannose via McpA. When key amino acids of McpA were mutated to alanine, the resulting point mutant strains showed chemorepulsion to these three compounds. For example, Y202 is associated with chemorepulsion to glutamate; R137, Y242, and T266 are associated with the avoidance of citric acid, and Y156, S160, N182, Y202, F204, and T242 are linked to the elimination of mannose [30]. Chemoreceptors are closely arranged on the cell membrane, where they may interact not only with downstream chemotactic regulatory proteins but also with other MCPs encoded within the genome. This close interaction may lead to changes in the overall chemotaxis to ligands following the mutation of key amino acids. Additionally, this process may result from the recognition of compounds by the chemoreceptor, which can detect signals not only in the periplasmic space but also in its other domains.

**Table 1:** Chemorepellents of bacteria.

Bacteria	Chemorepellents	MCPs	References
*Escherichia coli*	Low concentration of indole (below 1 mM)	Tsr	[64]
	Low concentration of dopamine (below 1 mM)	Tsr	[21]
	Melatonin and spermidine	Trg	
	High concentration of protocatechuic acid (above 50 μM)	Tar, Tsr	[21,62]
	High levels of norepinephrine (above 1 mM)	Tsr	
	1-Aminocyclohexane carboxylic acid	Tsr	[63]
*Pseudomonas*	Acids, bases, salts, and alcohols	unknown	[55]
	Hydrocarbons and heavy metals etc.	unknown	[56]
	Trichloroethylene, tetrachloroethylene, chloroform, dichloromethane, and other chemical reagent pollutants	unknown	[58]
*Bacillus velezensis*	Tert-butyl hydroxycarbamate	McpA	[20]
	Salicylic acid and sodium caprate	McpB	
	Dithiothreitol (DTT)	TlpA	
	Pentadecylic acid	TlpB	
*Campylobacter jejuni*	Succinic acid, thiamine, glucosamine, arginine, and lysine	CcmL	[31,32]
*Helicobacter pylori*	Six bile acids (taurocholic acid, taurodeoxycholic acid, glycholic acid, glycine deoxycholic acid, glycine deoxycholic acid, taurocholic acid) and two amino acids (aspartate and glutamic acid)	unknown	[67]
	AI-2	TlpB	[71]
	Low pH	TlpB	[72]
	Reactive oxygen species (ROS)	TlpD	[73]
*Vibrio cholerae*	D-Arg and D-Asp	MCP VC1313	[78]
*Salmonella typhimurium*	Phenol	unknown	[53]
	Insect repellent	unknown	[57]
*Rhodospirillum rubrum*	Some amino acids and salts	unknown	[54]
*Ligilactobacillus agilis*	Low pH, organic acids, bile salts, etc.	Mcp2	[74]

## 4. Conclusions and Prospects

In 1960, Adler first systematically studied the chemotactic mechanism of *E. coli*. Since then, chemotaxis has been studied for more than 60 years. Krell has summarized the sensory repertoire of bacterial chemoreceptors, aiming to establish relationships between LBD types, the nature of signals, and the mechanisms of signal recognition. Many in-depth and systematic studies on different microorganisms and different chemoeffectors have been conducted to explain the mechanisms underlying bacterial motility. There are both similarities and differences in the molecular mechanisms of chemotaxis among different bacteria. Notably, while the regulatory pathway is relatively conserved—where signals are transmitted to the flagella through the two-component regulatory system CheA-CheY to control flagellar rotation—the types and quantities of MCPs and the molecular mechanisms regulating adaptability vary widely among bacteria. Chemotaxis plays an important role in the colonization of rhizosphere bacteria, with increasing number of studies showing that plant roots attract beneficial bacteria to the rhizosphere by secreting root exudates. In general, amino acids and organic acids in root exudates are the primary effectors of bacterial chemotaxis.

As chemotaxis has advanced, several challenges have emerged. Firstly, differences in chemotaxis exist even among bacteria of the same genus but different species, necessitating the systematic study of each bacterium’s chemotaxis. Secondly, with the vast array of potential chemoeffectors in nature, rapidly screening these chemoeffectors presents a significant challenge that could impede progress in chemotaxis. Current methods involve qualitative experiments to systematically identify chemotactic effectors, quantitative chemotactic experiments are used to determine phenotypes, and isothermal titration experiments to explore interactions between proteins and small molecules. However, these methods are time-consuming, prone to false positives, and often inefficient for researchers. Lastly, chemoreceptors are the key receptors for binding chemoeffectors, making their structural study essential. Comparing chemoreceptor structures and predicting the small molecules they may bind could provide valuable insights for rapid screening. However, the binding between chemoreceptors and chemoeffectors depends on the LBDs. Unfortunately, more than 80 types of LBDs exist in nature, and research on rare LBDs is limited, hindering the development of repellent studies.

Bacteria show chemorepulsion to certain signal substances, such as fatty acids, fatty alcohols, amino acids, indoles, aromatic compounds, inorganic ions and mercaptans, most of which are toxic to cells. Previous studies have shown that some atypical chemoreceptors (where ligands bind to MCPs indirectly) may interact with downstream chemoreceptors to shift their homeostasis from the off state to the on state. This interaction is markedly different from the direct interaction observed with typical chemotactic ligands, which bind directly to the signal recognition domain of chemoreceptors. However, the molecular mechanism underlying the responses to atypical stimulus signals remain unknown.

The following questions should be considered in current and future studies. (i). Which compounds can act as chemorepellents for bacteria? (ii) What is the connection between hazardous compounds and chemorepellents? (iii) How can chemorepellents be quickly and accurately detected? (iv) Is there an intrinsic relationship between chemorepulsion and chemotaxis? (v) When both chemoattractants and chemorepellents are present in the environment, do bacteria cope with “conflicts” and respond?

## Data Availability

Data will be made available on request.
